# Traits of the Leaf Economics Spectrum Do Not Always Relate to Species Biomass Proportions in Grassland Communities of Varying Diversity

**DOI:** 10.1002/ece3.72013

**Published:** 2025-09-11

**Authors:** Dörte Bachmann, Nina Buchmann, Anne Ebeling, Christiane Roscher

**Affiliations:** ^1^ Institute of Agricultural Sciences ETH Zurich Zurich Switzerland; ^2^ Institute of Ecology and Evolution Friedrich Schiller University Jena Jena Germany; ^3^ Department of Physiological Diversity UFZ, Helmholtz Centre for Environmental Research Leipzig Germany; ^4^ German Centre for Integrative Biodiversity Research (iDiv) Halle‐Jena‐Leipzig Leipzig Germany

**Keywords:** biodiversity, functional traits, grassland, trade‐off, trait variation

## Abstract

Traits of the “leaf economics spectrum” (LES) relate to a functional trade‐off between slow and fast return on carbon investment, but it is not clear whether the globally observed trait‐relationships also hold in local plant communities. We studied four leaf traits associated with the LES in 20 grassland species, which represented different strategies in spatial resource acquisition (from small to large) and temporal resource acquisition (from early to late). Species were grouped into three partly overlapping pools of eight species to create a field experiment with 138 communities of varying species richness (1, 2, 3, 4, and 8 species), which either varied in spatial resource acquisition, temporal resource acquisition or both. Leaf nitrogen concentrations (N_Leaf_) and greenness (LeafG) decreased with species richness, while leaf dry matter content (LDMC) and specific leaf area (SLA) did not. Species with different spatial resource acquisition varied in leaf traits, suggesting that large species had “fast” traits (low LDMC, high N_Leaf_, dark LeafG), while small species had “slow” traits (high LDMC, low N_Leaf_, light LeafG). The extent of intraspecific trait variation (ITV) was smallest in LDMC and largest for N_Leaf_, but for all leaf traits, a greater portion of trait variation among communities was explained by interspecific trait variation (i.e., different species compositions) than ITV. Species with “fast” traits generally contributed more to total biomass than those with “slow” traits, but species biomass proportions did not always match expectations for all traits related to the “fast‐slow” trade‐off. Multi‐trait analyses showed that trait relationships expected from LES were not always present, and were modified by ITV and trade‐offs. In summary, independent responses of individual LES traits to local plant diversity lead to context‐dependent trait–trait relationships, which cannot easily be interpreted as differences in growth strategies, and thus limiting the general applicability of LES in grassland communities.

## Graphical abstract

We studied traits of the “leaf economics spectrum” (LES) in 138 experimental grassland communities composed of 20 grassland species, which represented different strategies in spatial resource acquisition (from small to large) and temporal resource acquisition (from early to late). We found that individual LES traits show independent responses to local plant diversity which lead to context‐dependent trait‐trait‐relationships and differences in the predictability of species biomass proportions in mixtures. We conclude that the general applicability of LES is limited in local grassland communities.

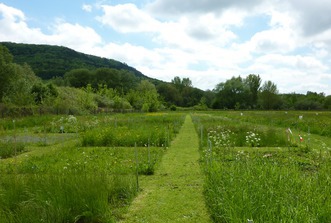

## 1 Introduction

One major research issue in community ecology is to understand processes and assembly rules explaining the coexistence of multiple plant species and mechanisms underlying plant community diversity. In recent years, trait‐based approaches offering the opportunity to generalize and predict ecological “strategies” of plant species have gained increasing attention (Lavorel and Garnier 2002; Shipley et al. 2016). A core assumption of trait‐based ecology is that realized combinations of traits are limited and shaped by different physiological or evolutionary trade‐offs and constraints. Easily measurable leaf traits, such as specific leaf area (SLA) and leaf nitrogen concentrations per mass (N_Leaf_) are integral parts of the so‐called “leaf economics spectrum” (LES), which is among the most prominent examples describing fundamental ecological relationships based on plant traits globally (Reich et al. 1997; Wright et al. 2004). Correlations among these traits describe a set of trade‐offs related to the construction and the maintenance costs of leaves and the plant carbon balance, which spans from “slow” plant species producing leaves with a “conservative” use of resources and slow carbon capture (i.e., low SLA, low N_Leaf_, low rates of photosynthesis, but a long leaf life span) to “fast” plant species with an “acquisitive” use of resources and fast carbon capture (i.e., high SLA, high N_Leaf_, high rates of photosynthesis, but a short leaf life span). The leaf economics spectrum (LES) is empirically based on global trait–trait correlations across multiple species (Wright et al. 2004; Shipley et al. 2006).

More recently, it has been questioned whether these global‐scale trait dimensions are also present at smaller ecological or taxonomic scales, which are more relevant for species interactions and processes of community assembly (Wright and Sutton‐Grier 2012; Funk and Cornwell 2013; Messier et al. 2017). Apart from the scale dependency, it is also well known that the expression of traits associated with the LES, such as SLA, LDMC, or N_Leaf_, may be controlled by local environmental conditions such as light and nutrient availability (e.g., Evans and Poorter 2001; Donovan et al. 2014; Siebenkäs et al. 2015; Firn et al. 2019), or local plant diversity (e.g., Gubsch et al. 2011; Roscher et al. 2011; Lipowsky et al. 2015). Previous studies have also shown that these traits vary over the growing season (Roscher et al. 2011; Bachmann et al. 2018). A recent meta‐analysis showed that values of SLA, LDMC, and N_Leaf_ differ between monocultures and mixtures, while the magnitude and direction of leaf trait responses to plant diversity are highly context dependent (Felix et al. 2023). The largest absolute effects were found for an increase of SLA with increasing species richness, while the effects were smaller and less consistent for N_Leaf_ and LDMC. The authors emphasized that studies addressing the effects of neighborhood diversity on leaf trait expression of herbaceous species are underrepresented in the literature and that a deeper understanding of neighborhood diversity effects on leaf traits should account for functional diversity within different species mixtures. Moreover, it remains an open question to which degree the extent of intraspecific variation in a certain LES trait in response to plant diversity is independent from variation in other LES traits, thereby changing trait–trait relationships and trade‐offs associated with the LES at the local scale.

It is reasonable that the abundance of plant species should be predictable from their traits in particular environments, but there are different hypotheses about how the distribution of trait values in a plant community could be related to species abundances. Community‐level trait composition is determined by interspecific trait differences, that is, identity and the relative abundances of species with different traits, and intraspecific trait variation (Lepš et al. 2011). The theory of limiting similarity assumes that species with different traits use different resources, thereby minimizing interspecific competition, which allows them to coexist (MacArthur and Levins 1967). However, interspecific trait differences may also reflect fitness differences, that is, species with particular traits may have a competitive advantage, resulting in competitive hierarchies and dominance of these species (Grime 2006; Herben and Goldberg 2014). Species with tall stature and leaf traits associated with fast growth are expected to be superior in competition and reach higher abundances at productive sites (Grime 2006; Reich 2014). However, given that the extent of intraspecific trait variation may vary considerably between different traits and different species and the uncertainty that traits vary in a coordinated way in response to local plant community diversity, trait‐abundance relationships may vary locally, limiting the general application of the LES.

Here, we studied patterns of intra‐ and interspecific leaf trait variation and tested how they were affected by local plant diversity and related to species abundance within the studied plant communities. We conducted the study in the so‐called Trait‐Based Biodiversity Experiment (TBE) (Ebeling et al. 2014), which is part of a long‐term grassland biodiversity experiment (Jena Experiment; Roscher et al. 2004). The TBE simultaneously manipulates both plant species richness and functional diversity. Using whole‐plant traits related to spatial and temporal acquisition of resources, the TBE represents gradients in plant species richness within three partly overlapping pools of species that vary (1) in their spatial resource acquisition traits (as related to the size of the plants and their organs), (2) in their temporal resource acquisition traits (as related to plant phenology), or (3) in a mix of both to create different scenarios of non‐random species loss. Each species pool consists of eight plant species, and each plant species occurs in mixtures of each species richness level, making the experiment particularly suitable for the study of intra‐ and interspecific trait variation. We measured four leaf traits, two of which are key traits of the LES (SLA and N_Leaf_), and two others which also relate to aboveground resource acquisition (LDMC, and leaf greenness as surrogate for chlorophyll content) and are easy to measure for a large number of samples. We asked the following questions: (1) do leaf traits associated with the LES show a coordinated response to increasing plant diversity? (2) are between‐species trait–trait relationships consistent among species pools and are within‐species correlations of LES traits consistent across species? (3) do species which vary in whole‐plant traits related to spatial and temporal resource acquisition differ in their extent of intraspecific leaf trait variation and does intraspecific trait variation explain a significant share of trait variation among local plant communities? (4) do species with leaf traits associated with fast carbon capture (high SLA, high N_Leaf_, dark leaf greenness, low LDMC) reach higher biomass proportions in plant mixtures than species with low carbon capture traits?

## 2 Material and Methods

### 2.1 Experimental Design

The Jena Experiment started in 2002 (Roscher et al. 2004). The experimental site is located in the floodplain of the river Saale, north of the city of Jena (Thuringia, Germany, 50°55′N, 11°35′E, 130 m a.s.l.). Mean annual temperature is 9.9°C, and mean annual precipitation is 610 mm (1980–2010; Hoffmann et al. 2014).

The site of the Jena Experiment harbors various experiments. Here, we used the Trait‐Based Biodiversity Experiment (TBE), which was established in 2010 (for detailed description, see Ebeling et al. 2014). Briefly, 20 non‐legume plant species were identified with a principal components analysis (PCA) from the non‐legume species of the sown species pool of the Jena Experiment (comprising 48 non‐legume species and 60 species in total; Roscher et al. 2004) to represent a gradient with respect to plant‐size traits (leaf size, plant height, rooting depth, and root length density) associated with spatial resource acquisition from “small” to “large” species (PC1) and with respect to phenology traits (growth starting date, flowering starting date) associated with their temporal resource acquisition from “early” to “late” species (PC2). The traits used for the PCA were measured in species monocultures of the Jena Experiment. Based on these two dimensions of trait variation, three partially overlapping species pools were defined, each comprising eight plant species. The species pools were selected for their variation in specific trait combinations. The pool “SpatRes” covered the whole range of trait variation with respect to spatial resource acquisition (PC1) without variations along PC2, that is, these species were similar in their temporal resource acquisition. The pool “TempRes” represented the whole range of PC2 with little variation along PC1, that is, these species varied mostly in their temporal resource use but showed little variation in their spatial resource acquisition. Finally, the pool “MixRes” represented the extremes in spatial and temporal resource use in the total species pool (for a list of plant species, their assignment to species pools and positions on PC1 and PC2, see Table S1). From each species pool, 46 plant communities of different species richness (1, 2, 3, 4 and 8 species) and functional diversity in spatial and/or temporal resource acquisition (FD_Spatial_, FD_Temporal_) were assembled, resulting in a total of 138 plant communities (see https://jexis.idiv.de/ddm/data/Showdata/425 for species compositions), which were sown on plots of 3.5 × 3.5 m size. Each species occurred between 14 to 16 times in the plant communities of its species pool (Table S1). In the mixtures, all species were sown with equal proportions, i.e., the total number of sown seeds (=1000 seeds per m^2^) was divided by the number of species. The experimental plots were weeded three times per year and mown twice per year. No fertilizer was added.

### 2.2 Data Collection

Leaf traits were measured for all species available in each plot twice during the growing season at peak biomass shortly before mowing: from 28 to 30 May 2012 and from 26 to 27 August 2012. Leaf greenness (LeafG; unitless) was measured on a young, but fully expanded leaf of three individual shoots per species in each plot with a portable chlorophyll meter (SPAD‐502, Konica‐Minolta, Osaka, Japan). The chlorophyll meter measures the transmittance of the leaf at two wavelengths (650 nm and 940 nm), resulting in dimensionless values. Because of their narrow leaves, LeafG could not be measured for *Festuca rubra* (all plots) and *Poa pratensis* (five plots) in May 2012. Values of leaf greenness were averaged to get a species‐level mean value per plot and season.

Afterwards, bulk samples of 5 to 10 young and fully expanded leaves per species were sampled in each plot, put into moist tissue paper, and stored at 4°C overnight in sealed plastic bags for rehydration. Then, the fresh weight of leaf samples was determined after removing any water droplets with tissue paper. Subsequently, leaf area was measured using a leaf area meter (LI‐3100, LICOR, Lincoln, USA). All samples were dried at 70°C for 48 h and weighed again to obtain the dry weight. Specific leaf area (SLA; mm^2^ mg^−1^) was calculated as the ratio of leaf area to dry weight, and leaf dry matter content (LDMC; mg g^−1^) as the ratio of dry weight to fresh weight. Leaf samples were ground with a mixer mill (MM200, Retsch, Germany) and analyzed with an elemental analyzer (FlashEA 112, Thermo Electron, Italy) to obtain leaf nitrogen concentrations per mass (N_Leaf_; mg N g^−1^).

Aboveground biomass was harvested in spring (29 May to 4 June 2012) and summer (27 to 31 August 2012) at estimated peak biomass before mowing. Plants were cut 3 cm above the soil surface within two randomly allocated subplots of 0.5 × 0.2 m size in each plot (Wagg et al. 2017). Samples were sorted by sown species, detached dead plant material, and weeds, and then dried at 70°C for 48 h before weighing. Species biomass was derived as means from the two replicated samples per plot.

### 2.3 Statistical Analyses

Data were analyzed with the statistical software R 4.2.3 (R Core Team 2023). First, we applied linear mixed‐effects models with the function *lmer* in the R library *lme4* (Bates et al. 2015) to evaluate whether traits associated with the LES show coordinated responses to changes in plant diversity (question 1). Fixed effects comprised plant diversity as species richness (SR; as log‐linear term), functional diversity in spatial resource acquisition (FD_Spatial_), functional diversity in temporal resource acquisition (FD_Temporal_), species positions along dimensions of spatial (STG) and temporal (TTG) resource acquisition traits, and the interactions between plant diversity and a species' position regarding the dimensions of spatial or temporal resource acquisition (SR × STG, SR × TTG). FD_Spatial_ and FD_Temporal_ for each community were defined as the average pairwise species distances in traits associated with spatial or temporal resource acquisition, using the species scores of the first or second principal component, respectively (see Table S1). STG and TTG were the scores of individual species on principal components axis 1 (STG) and principal components axis 2 (TTG). Furthermore, we included species pool (Pool; factor with three levels: SpatRes, TempRes, MixRes) and the interactions of pool with plant diversity (Pool × SR) to test how plant diversity effects on leaf trait expression depended on the species pool from which the communities were assembled. Finally, we added season and the respective interactions to test if season influenced the effects of plant and functional diversity on leaf trait expression (Season × SR, Season × FD_Spatial_, Season × FD_Temporal_) and if seasonal variation in leaf trait expression depended on species resource‐acquisition traits (Season × STG, Season × TTG, Season × Pool). Fixed effects were added stepwise, starting from a constant null model with block and plot (nested in block) and species identity as independent random effects. The maximum likelihood method and likelihood ratio tests (*χ* ^2^ ratio) for model comparisons were used to evaluate the significance of the fixed effects.

To evaluate how consistent the leading axes of trait variation and patterns of trait–trait relationships were between and within species (question 2), we conducted standardized principal components analyses (PCAs) on leaf trait data for both seasons using the R library *ade4* (Dray and Dufour 2007). First, we extracted the leading axes of trait variation for each pool using species trait means across all plots (between‐species PCA). Second, we used the deviation of species trait means per diversity level from species trait means across all plots; third, we used the deviation of species plot‐level data from species trait means per diversity level to assess the effects of plant diversity and the effects of species compositions within diversity levels on trait dimensions. Finally, we analyzed traits of each species separately to assess the consistency of trait patterns at the intraspecific level (i.e., axes discriminating samples of the same species collected on different plots).

To assess whether species varying in whole‐plant traits related to spatial and temporal resource acquisition differed in the extent of ITV, that is, species‐level trait variation between plots (question 3), we calculated the coefficient of variation (CV) by dividing the mean of plot‐level trait values by the standard deviation of plot‐level trait values for each species separately per pool and season. Afterwards, we used the CV as a response variable in a linear mixed‐effects model with trait (four factor‐levels), species positions along the gradients of spatial and temporal resource acquisition (STG, TTG), pool, season, and pairwise interactions between these explanatory variables as fixed effects and species identity as a random effect. Posthoc comparisons were done using the R library *emmeans* (Lenth 2024). To quantify the relative contribution of species composition and ITV to community‐level changes in leaf traits, we used the partitioning method proposed by Lepš et al. (2011). Briefly, we calculated plot‐level abundance‐weighted community means, using either the species‐specific trait values measured within a particular plot (CWM_total_) or averaged across all plots of a species pool for each season (CWM_fixed_) and species biomass proportions in the mixtures as a measure of relative abundance. The intraspecific variability effect was calculated as the difference between both CWMs (CWM_intra_). The sum of squares associated with CWM_total_, CWM_fixed_ and CWM_intra_ across all communities and both seasons (SS_total_, SS_fixed_ and SS_intra_) were calculated using an intercept‐only linear model. SS_total_ is the total variation in CWM between communities, SS_intra_ represents variation due to intraspecific variability, and SS_fixed_ represents variation due to differences in species occurrence and relative abundances.

To test if species with leaf traits associated with fast carbon capture reached higher biomass proportions (question 4), we calculated the ratio ((CWM/CM)‐1) based on community mean traits weighted by species biomass proportions (CWM) and simple community trait means based on species presence‐absence (CM) (Siebenkäs et al. 2017). Cases with ((CWM/CM)‐1) not significantly different from zero either had similar trait values of species, irrespective of their biomass proportions, or realized abundances in a mixture were equal for all species in a mixture. Cases for which ((CWM/CM)‐1) was significantly different from zero indicated that trait values of more dominant species were larger ((CWM/CM)‐1 > 0) or smaller ((CWM/CM)‐1 < 0) than those of less abundant species. To test if trait deviations of more abundant species varied dependent on species richness, pool, or season, we applied linear‐mixed effects models with these terms and their interactions as fixed effects. Block and plot (nested block) were incorporated as random effects.

## 3 Results

### 3.1 Leaf Trait Responses to Changes in Diversity and Community Composition

**FIGURE 1:**
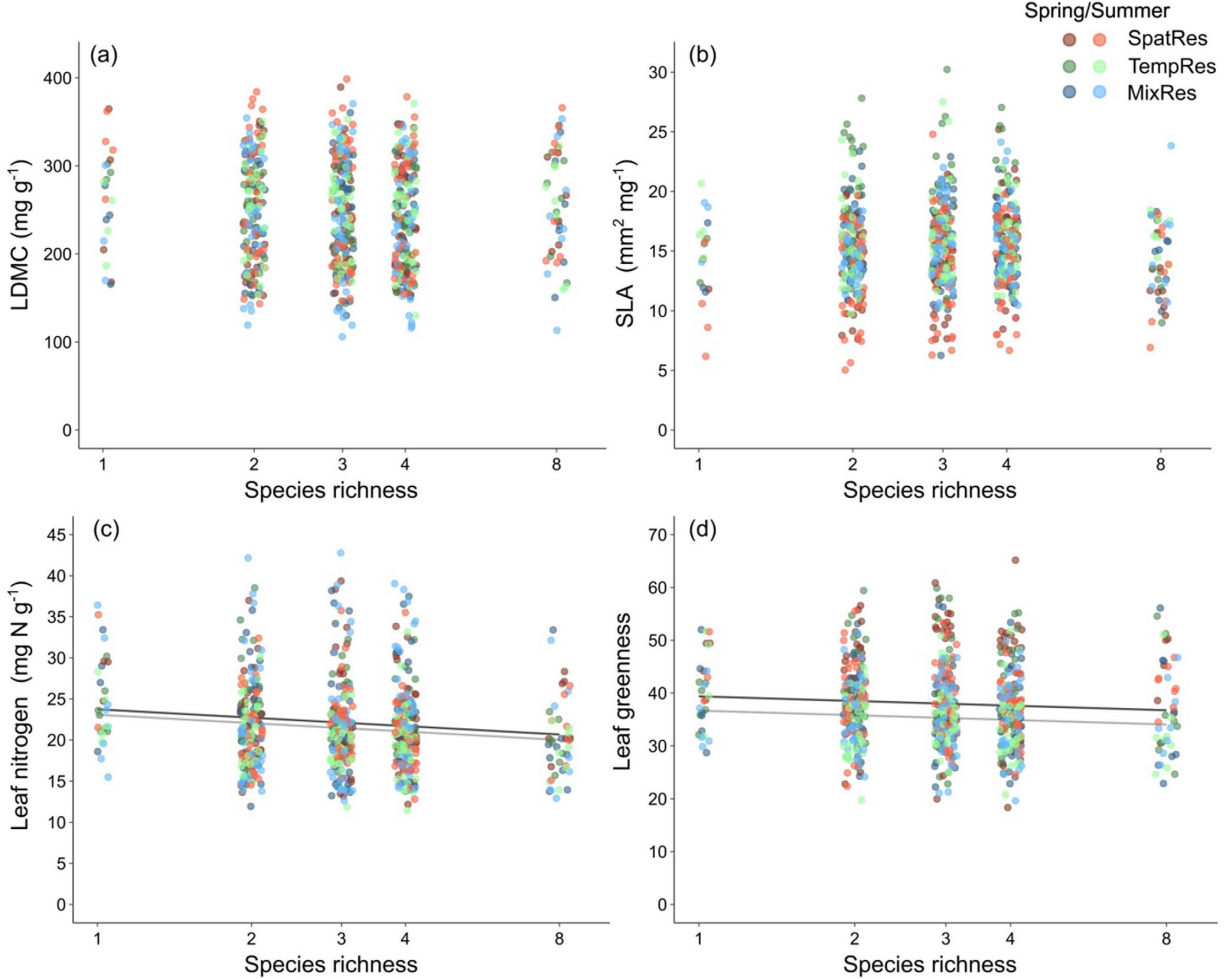
Leaf dry matter content (LDMC) (a), specific leaf area (SLA) (b), leaf nitrogen concentration (N_Leaf_) (c), and leaf greenness (LeafG) (d) related to species richness (log‐scale). Different symbols indicate species assignment to the experimental species pools (see Table S1), darker colors represent spring and lighter colors summer. Relationships were analyzed in linear mixed‐effects models (Table 1); regression lines indicate the slope and significance of the relationships (black = spring, gray = summer).

**FIGURE 2:**
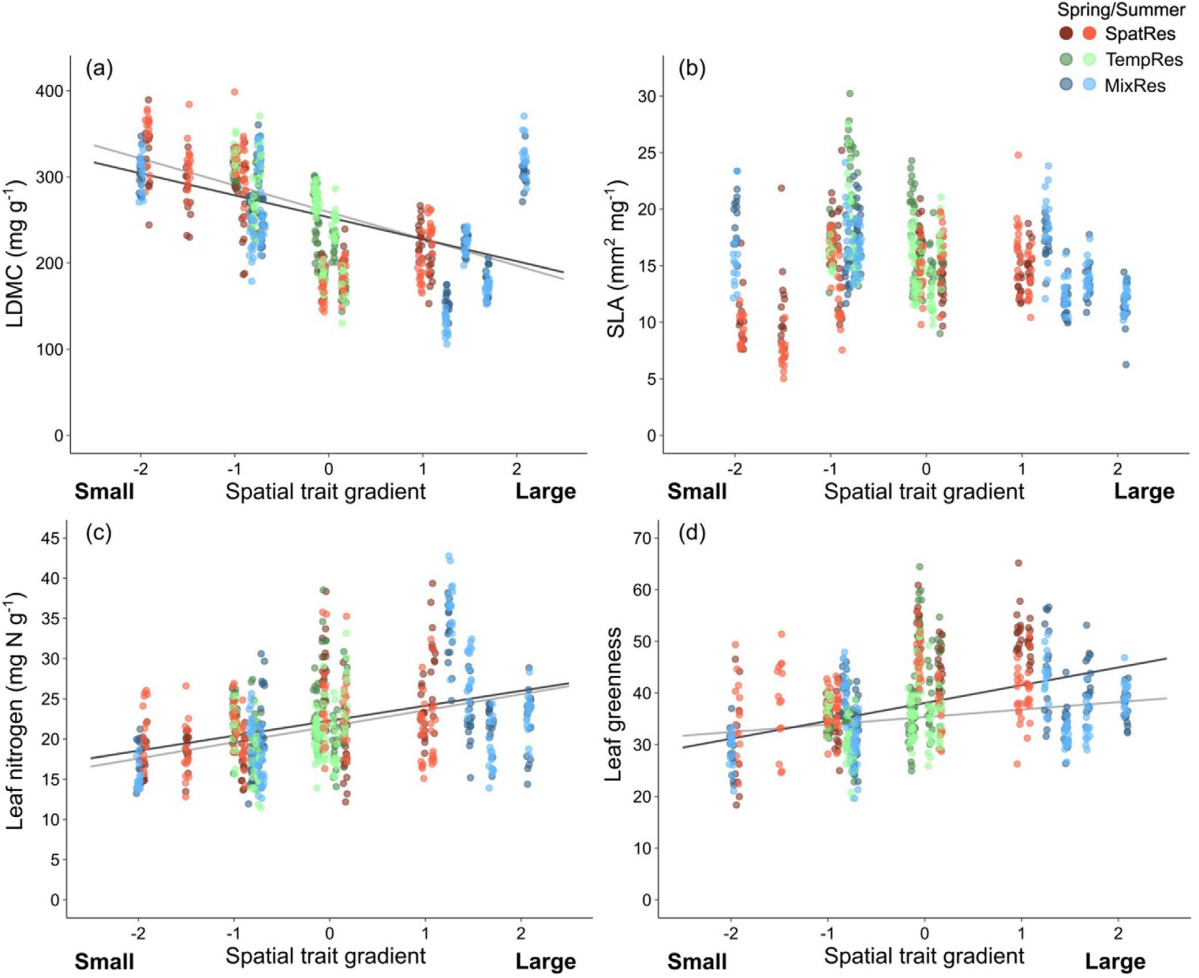
Leaf dry matter content (LDMC) (a), specific leaf area (SLA) (b), leaf nitrogen concentration (N_Leaf_) (c), and leaf greenness (LeafG) (d) related to the spatial trait resource acquisition gradient. Different symbols indicate species assignment to the experimental species pools (see Table S1); darker colors represent spring and lighter colors summer. Relationships were analyzed in linear mixed‐effects models (Table 1); regression lines indicate the slope and significance of the relationships (black = spring, gray = summer).

Plant diversity, that is, species richness (SR), did not affect leaf dry matter content (LDMC) and specific leaf area (SLA), but led to a decrease in leaf nitrogen concentrations (N_Leaf_) and leaf greenness (LeafG) (Table 1 and Figure 1). Plant community diversity in spatial or temporal resource acquisition did not affect leaf traits (Table 1). Species representing different positions along the spatial resource‐acquisition gradient (STG) differed in their leaf traits: LDMC declined, while N_Leaf_ and LeafG increased from “small” to “large” species. SLA varied independently along the STG (Table 1 and Figure 2). Species representing different positions along the temporal resource‐acquisition gradient (TTG) did not show clear patterns in leaf traits between “early” and “late” species (Table 1 and Figure S1). However, the strengths of negative species‐richness effects on N_Leaf_ and LeafG were less pronounced in “late” than in “early” species (significant interaction TTG × SR; Table 1), while the strength of negative species‐richness effects on LeafG was stronger for “small” than for “large” species (significant interaction STG × SR; Table 1). Species pools assembling species along dimensions of spatial and temporal resource acquisition traits did not differ in leaf traits, with the exception of larger LDMC values in the mixed pool representing the extremes in spatial and temporal resource acquisition (MixRes; Table 1).

**TABLE 1:** Summary of linear mixed‐effects model analyses for leaf dry matter content (LDMC), specific leaf area (SLA), leaf nitrogen concentration (N_Leaf_), and leaf greenness (LeafG) as affected by plant community diversity (SR, FD_Spatial_, FD_Temporal_) and species differences in spatial and temporal resource acquisition (STG, TTG), pool, and season.

Source of variation	df	LDMC			SLA		
		*χ* ^2^	p		*χ* ^2^	*p*	
Species richness (SR)	1	0.423	0.515		0.065	0.799	
FD_Spatial_	1	1.733	0.188		2.891	0.089	
FD_Temporal_	1	0.044	0.834		0.655	0.418	
Spatial trait gradient (STG)	1	**8.356**	**0.004**	↓	0.153	0.696	
Temporal trait gradient (TTG)	1	1.466	0.226		0.330	0.566	
SR × STG	1	1.862	0.172		1.105	0.293	
SR × TTG	1	0.520	0.471		0.714	0.398	
Pool	2	**7.021**	**0.030**		2.505	0.286	
SR × Pool	2	0.618	0.734		3.423	0.181	
Season	1	**15.946**	** < 0.001**		**22.347**	** < 0.001**	
Season × SR	1	0.093	0.760		3.423	0.064	
Season × FD_Spatial_	1	**6.555**	**0.010**		**6.287**	**0.012**	
Season × FD_Temporal_	1	0.001	0.977		0.003	0.956	
Season × STG	1	**13.154**	** < 0.001**		**36.812**	** < 0.001**	
Season × TTG	1	0.684	0.408		2.448	0.118	
Season × Pool	2	**25.009**	** < 0.001**		5.966	0.051	

Source of variation	df	N_Leaf_			LeafG		
		*χ* ^2^	*p*		*χ* ^2^	*p*	
Species richness (SR)	1	**22.834**	** < 0.001**	↓	**13.519**	** < 0.001**	↓
FD_Spatial_	1	0.026	0.871		0.124	0.725	
FD_Temporal_	1	1.387	0.239		0.185	0.667	
Spatial trait gradient (STG)	1	**8.431**	**0.004**	↑	**5.034**	**0.025**	
Temporal trait gradient (TTG)	1	2.713	0.100		0.002	0.969	
SR × STG	1	0.212	0.645		**5.071**	**0.024**	
SR × TTG	1	**6.255**	**0.012**		**5.416**	**0.020**	
Pool	2	0.106	0.949		0.977	0.614	
SR × Pool	2	0.906	0.636		0.878	0.645	
Season	1	**5.805**	**0.016**		**46.174**	** < 0.001**	
Season × SR	1	0.083	0.773		0.849	0.357	
Season × FD_Spatial_	1	**7.538**	**0.006**		0.094	0.759	
Season × FD_Temporal_	1	0.500	0.480		1.282	0.258	
Season × STG	1	0.267	0.605		**29.757**	** < 0.001**	
Season × TTG	1	0.340	0.560		**4.428**	**0.035**	
Season × Pool	2	**6.481**	**0.039**		**23.102**	** < 0.001**	

*Note:* Models were fitted by stepwise inclusion of fixed effects. Listed are the results of likelihood ratio tests (*χ* ^2^) that were applied to assess model improvement and the statistical significance of the fixed effects (*p*‐values). Significant effects (*p* < 0.05) are marked in bold. Arrows indicate higher (↑) or lower (↓) trait values with increasing species richness or shifts along the spatial or temporal trait gradient.

All leaf traits differed between spring and summer. On average, SLA, N_Leaf_ and LeafG were larger, and LDMC was smaller in spring than in summer, but the extent and direction of seasonal trait variation differed dependent on plant community diversity in spatial resource acquisition (for LDMC, SLA and N_Leaf_), dependent on a species' position on the spatial resource acquisition gradient (for LDMC, SLA and LeafG; Figure 2) or the temporal resource acquisition gradient (for LeafG; Figure S1) and among species pools (Table 1).

### 3.2 Main Axes of Leaf Trait Variation

**FIGURE 3:**
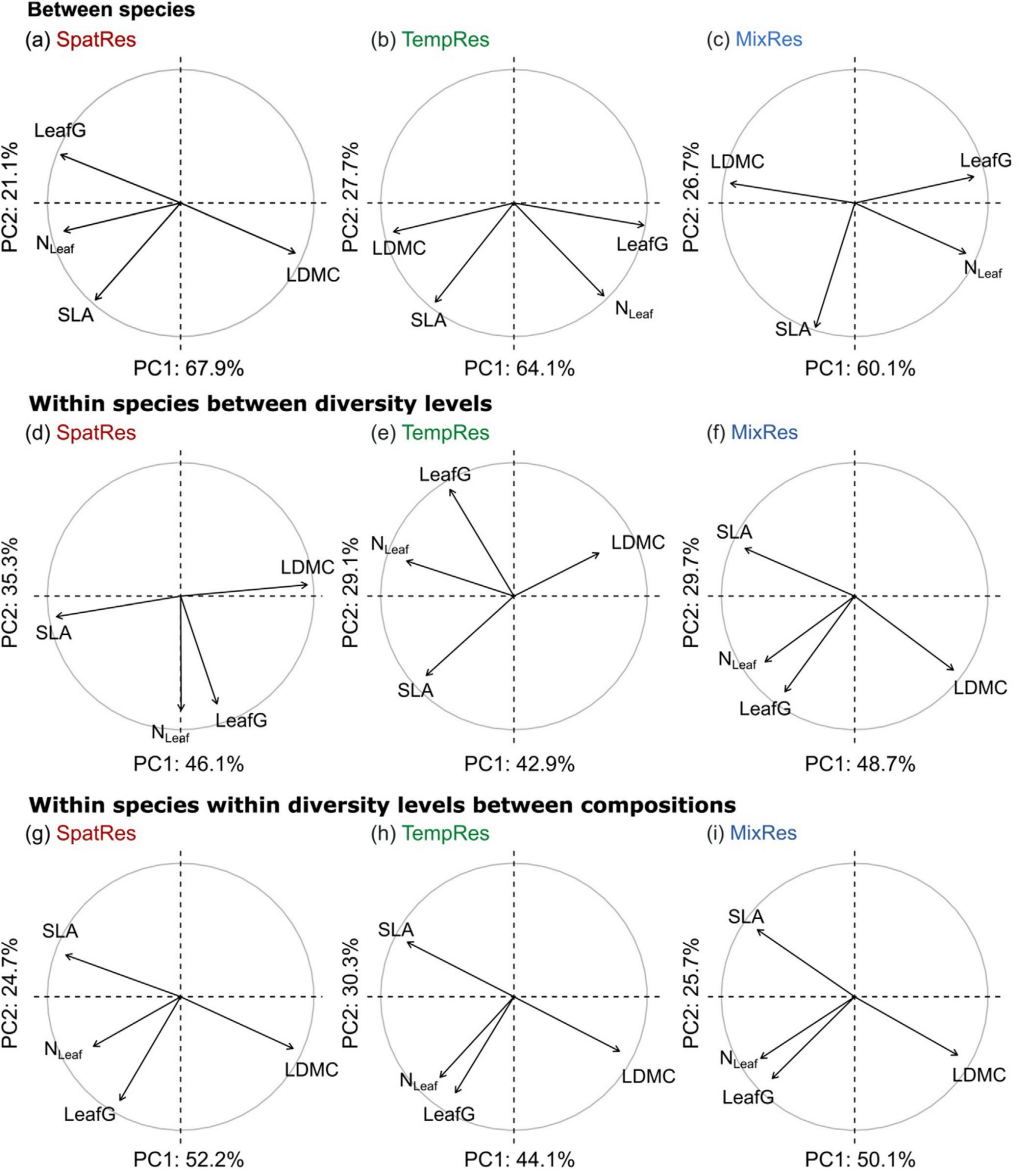
Standardized principal component analyses (PCA) of leaf traits measured in spring based on species mean traits across all communities (a–c), based on deviations of species trait means per diversity level from species trait means across all communities (d–f), and based on deviations of plot‐level species trait values from species trait means per diversity level (g–i). PCA was performed separately for each pool SpatRes (a, d, g), TempRes (b, e, h) and MixRes (c, f, i). Correlation circles based on the first two principal components and proportions of explained variation are given. Abbreviations of traits: LDMC = leaf dry matter content, LeafG = leaf greenness, N_Leaf_ = leaf nitrogen concentration, SLA = specific leaf area.

The PCA on between‐species variation (=interspecific variation) in leaf traits for each pool produced similar leading axes of variation and structures of trait space for the three species pools in both seasons (Figure 3a–c and Figure S2a–c). The first axis explained between 54% and 68% of the variation and showed high correlations with LDMC, which was opposed to LeafG and N_Leaf_. SLA was more related to the second axis, which explained between 21% and 28% of thevariation.

The PCA focusing on within‐species variation (=intraspecific variation) in leaf traits between diversity levels (Figure 3d–f and Figure S2d–f) or within diversity levels between different species compositions (Figure 3g–i and Figure S2g–i) also showed in most cases the same structure and leading axes, irrespective of species pools and seasons. Here, SLA and LDMC were opposed to each other. They either correlated strongly with the first axis or were located between the first and second axes, indicating that multi‐trait variation in response to diversity and community composition was mainly driven by changes in SLA and LDMC. Variations in N_Leaf_ and LeafG were positively related and often located near‐orthogonal to the variations in SLA and LDMC.

Separate analyses of individual species for spring and summer (Figures S3 and S4) also showed in many, but not all cases, that SLA and LDMC were negatively correlated and determined the first axis, while LeafG and N_Leaf_ were positively related and near‐orthogonally located on the second axis. However, for some species (e.g., *Plantago lanceolata* in spring, or *Anthoxanthum odoratum* in summer), multi‐trait variation was less reproducible; that is, SLA and N_Leaf_ showed closer relationships, and N_Leaf_ varied more independently from LeafG.

### 3.3 Extent of Intraspecific Variation vs. Interspecific Differences in Leaf Traits

The extent of ITV quantified as the coefficient of variation varied among leaf traits and decreased in the order N_Leaf_>SLA>LeafG>LDMC. While the extent of ITV in LDMC was significantly lowest, differences in ITV between other traits were mostly insignificant, except for higher ITV in N_Leaf_ than LeafG (Table 2 and Figure S5). The extent of ITV in leaf traits did not vary among species with different positions along the gradient of temporal resource acquisition. Along the gradient of spatial resource acquisition, however, “large” species showed a lower extent of ITV in SLA and LeafG than “small” species (significant interaction Trait × STG). Moreover, ITV was generally larger in the pool presenting species with different spatial resource acquisition (SpatRes) than in other pools, but at the level of individual traits, this was only significant for SLA (*p* = 0.001). The extent of ITV in leaf traits was not different between spring and summer.

**TABLE 2:** Summary of linear mixed‐effects model analysis for intraspecific trait variation (ITV) quantified as coefficient of variation (CV) as varying among traits and depending on species differences in spatial and temporal resource acquisition (STG, TTG), pool and season.

Source of variation	df	*χ* ^2^	*p*
Trait	3	**80.136**	** < 0.001**
Spatial trait gradient (STG)	1	2.796	0.094
Temporal trait gradient (TTG)	1	1.101	0.294
Pool	2	**11.276**	**0.004**
Trait × STG	3	**12.420**	**0.006**
Trait × TTG	3	5.490	0.139
Trait × Pool	6	6.520	0.367
Season	1	2.362	0.124
Season × Trait	3	4.001	0.261
Season × STG	1	0.760	0.383
Season × TTG	1	0.025	0.874
Season × Pool	2	2.385	0.303

*Note:* Models were fitted by stepwise inclusion of fixed effects. Listed are the results of likelihood ratio tests (*χ* ^2^) that were applied to assess model improvement and the statistical significance of the fixed effects (*p*‐values). Significant effects (*p* < 0.05) are marked in bold.

**FIGURE 4:**
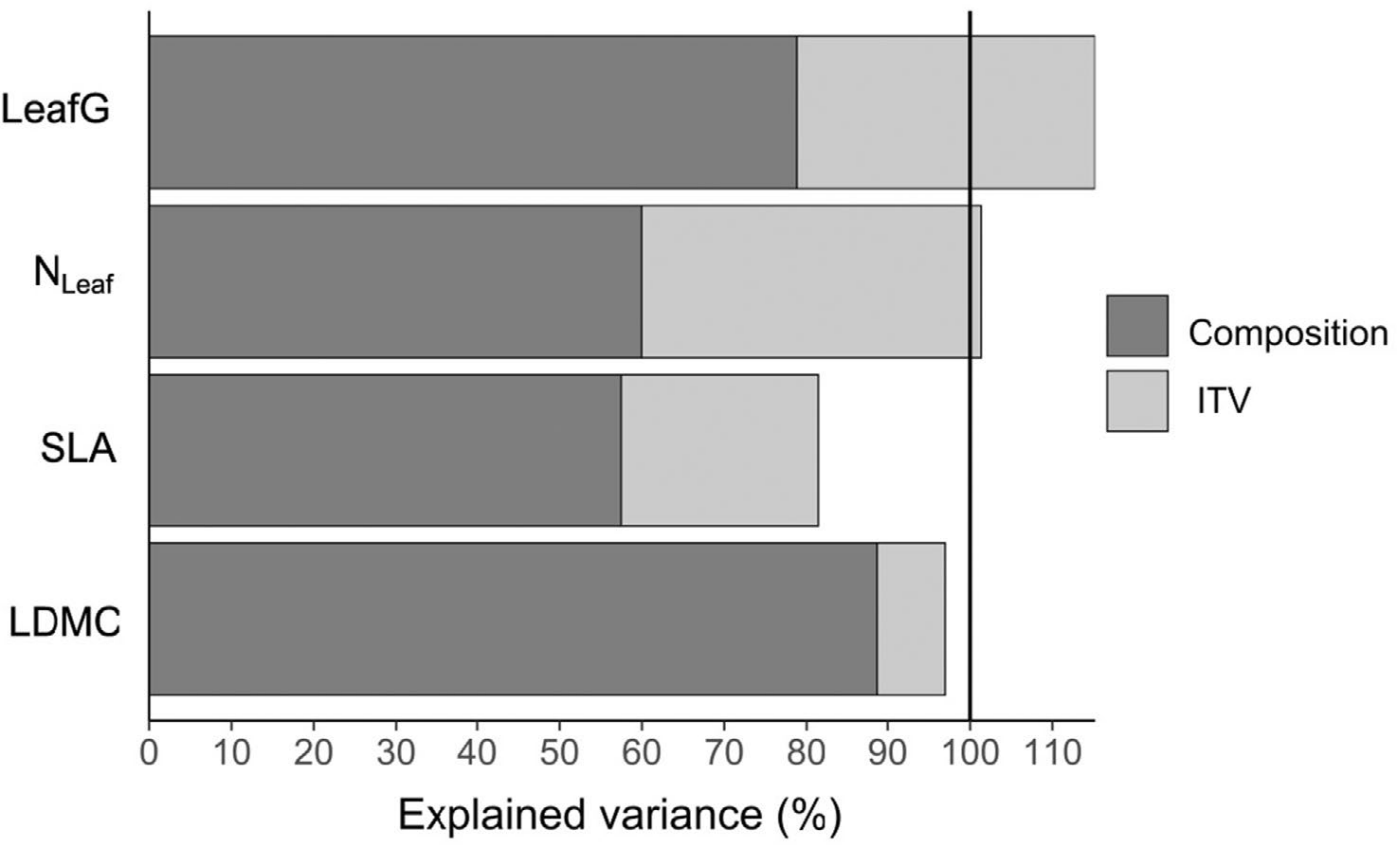
Variance decomposition of abundance‐weighted community trait means (CWM) into the contributions of species composition (dark gray part of the bars), and intraspecific trait variation (ITV; light gray part of the bars) across all plots and both seasons. The black line indicates total variation; space between the bars and the line corresponds to the effects of covariation, which is positive if the line is above the column and negative if the line crosses the column.

Overall, a larger portion of variation in community‐weighted trait means (CWM) of the plant mixtures was explained by different species compositions (accounting for 57%–89% of the explained variation) than by ITV (Figure 4). ITV explained only ~8% of variation for CWM of LDMC; ~24% of variation for CWM of SLA, and reached ~41% and 36% for CWM of N_Leaf_ and LeafG, respectively. For CWM of SLA, variation explained by species composition and ITV showed a large positive covariation (accounting for ~19% of variation), indicating that the occurrence and high abundances of species with high SLA and increasing values of SLA of these species jointly explained larger CWM. Covariation of species composition and ITV was also positive, but small for CWM of LDMC (accounting for ~3% of variation), while it was negative for CWM of N_Leaf_ (explaining 1% of variation) and CWM of LeafG (explaining 15% of variation). The negative covariation indicated that high abundances of species with large values in N_Leaf_ or LeafG were associated with lower species‐level values in these traits.

### 3.4 Leaf Traits in Relation to Species Biomass Proportions

**FIGURE 5:**
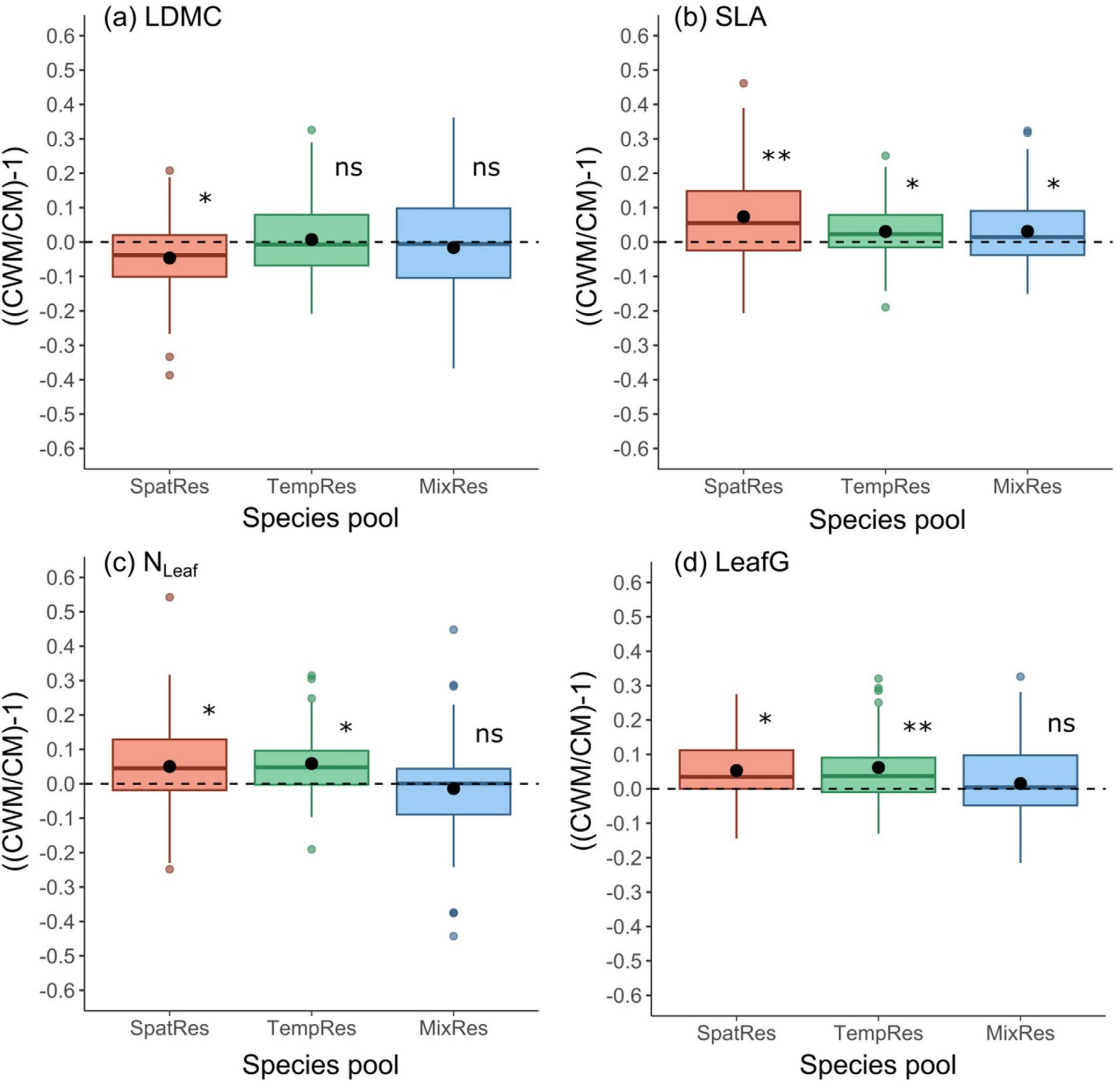
Ratios of abundance‐weighted community trait means over simple community trait means ((CWM/CM)‐1), which indicate differences in trait values between dominant and subordinate species, for leaf dry matter content (a), specific leaf area (b), leaf nitrogen concentration (c), and leaf greenness (d). Shown are boxplots for each species pool (SpatRes, TempRes, MixRes) across both seasons with median, interquartile range, maximum and minimum values, and outliers. Black dots indicate mean values per species pool. Significant deviations from zero across all species‐richness levels tested in linear mixed‐effects models are indicated for each species pool as *p < 0.05, **p < 0.01, ns = non‐significant. Ratios of ((CWM/CM)‐1) significantly different from zero indicate that trait values of dominant species are larger ((CWM/CM)‐1) > 0 or smaller ((CWM/CM)‐1) < 0 than those of subordinate species.

Differences in trait values dependent on species biomass proportions did not change with species richness (Table 3). Higher plant community diversity in spatial resource acquisition was associated with greater biomass proportions of species with large SLA and N_Leaf_, while differences in LeafG among species became smaller (Figure 5; Table 3). The extent of trait differences between dominants and subordinates varied among mixtures assembled from different species pools. In mixtures of species with different spatial resource acquisition traits (SpatRes), dominant species had smaller values in LDMC and larger values in SLA, N_Leaf_ and LeafG than subordinate species. In mixtures of species with different temporal resource acquisition traits (TempRes), dominant and subordinate species did not differ in LDMC, but SLA, N_Leaf_ and LeafG were also larger in dominant compared to subordinate species. In mixtures of species representing the extremes in spatial and temporal resource acquisition traits (MixRes), dominant species also had larger SLA than subordinate species, but they did not differ in LDMC, N_Leaf_ or LeafG.

**TABLE 3:** Summary of linear mixed‐effects model analyses for ratios of biomass‐weighted community means over simple community means ((CWM/CM)‐1) for leaf dry matter content (LDMC), specific leaf area (SLA), leaf nitrogen concentration (N_Leaf_), and leaf greenness (LeafG) as affected by plant community diversity (SR, FD_Spatial_, FD_Temporal_) pool, and season.

Source of variation	df	LDMC			SLA		
		*χ* ^2^	*p*		*χ* ^2^	*p*	
Species richness (SR)	1	0.136	0.713		1.826	0.177	
FD_Spatial_	1	0.122	0.727		**5.312**	**0.021**	↑
FD_Temporal_	1	0.023	0.880		0.149	0.699	
Pool	2	**6.431**	**0.040**		**9.107**	**0.011**	
Pool × SR	2	1.086	0.581		0.758	0.685	
Season	1	1.571	0.210		0.009	0.923	
Season × SR	1	0.339	0.561		2.126	0.145	
Season × FD_Spatial_	1	0.538	0.463		0.018	0.893	
Season × FD_Temporal_	1	0.129	0.720		0.030	0.862	
Season × Pool	2	1.803	0.406		3.442	0.179	

Source of variation	df	N_Leaf_			Leaf greenness		
		*χ* ^2^	*p*		*χ* ^2^	*p*	
Species richness (SR)	1	1.127	0.288		3.362	0.067	
FD_Spatial_	1	**3.999**	**0.046**	↑	**4.874**	**0.027**	↓
FD_Temporal_	1	0.041	0.840		0.125	0.724	
Pool	2	**12.880**	**0.002**		4.244	0.120	
Pool × SR	2	**6.110**	**0.047**		**6.277**	**0.043**	
Season	1	**12.166**	** < 0.001**		0.020	0.889	
Season × SR	1	0.157	0.692		0.242	0.623	
Season × FD_Spatial_	1	0.796	0.372		0.400	0.527	
Season × FD_Temporal_	1	**7.368**	**0.007**		0.783	0.376	
Season × Pool	2	3.281	0.194		0.970	0.616	

*Note:* Models were fitted by stepwise inclusion of fixed effects. Listed are the results of likelihood ratio tests (*χ* ^2^) that were applied to assess model improvement and the statistical significance of the fixed effects (*p*‐values). Significant effects (*p* < 0.05) are marked in bold. Arrows indicate a higher (↑) or lower (↓) ratio of ((CWM/CM)‐1) with increasing functional diversity.

## 4 Discussion

### 4.1 Leaf Traits in Response to Plant Diversity

We used the experimental design of the Trait‐Based Biodiversity Experiment to assess to which extent traits associated with the LES varied in response to plant diversity and community composition. A recent meta‐analysis by Felix et al. (2023) showed that the response of leaf traits to neighboring diversity was highly heterogeneous. In line with these results, we found that leaf traits differed in their responsiveness to plant diversity. While leaf traits associated with nutritional status (N_Leaf_, LeafG) decreased with increasing species richness, LDMC and SLA did not show systematic changes along the gradients of species richness. Previously, trait variation in response to plant diversity had been extensively studied in the main Jena Experiment (Roscher et al. 2018), which also contains legumes. In the main experiment, N_Leaf_ and LeafG did not change with increasing species richness, but the presence of legumes had positive effects on both traits in non‐legume species (Gubsch et al. 2011; Lipowsky et al. 2015; Bachmann et al. 2018). Furthermore, LDMC did not respond to species richness or the presence of legumes in the main experiment (Bachmann et al. 2018), which is consistent with the current study and the results of the meta‐analysis by Felix et al. (2023). Decreasing N_Leaf_ with higher species richness in the Trait‐Based Biodiversity Experiment could be due to more complete use of soil resources at higher plant diversity. Various biodiversity experiments have shown that especially in communities without legumes available soil nitrogen (nitrate) decreased at higher plant diversity and correlated negatively with plant biomass production (e.g., Palmborg et al. 2005; Roscher et al. 2008). The higher biomass production of more diverse communities, which has also been shown for the Trait‐Based Biodiversity Experiment (Wagg et al. 2017), might also cause a “dilution” of tissue N concentration (per mass) at peak biomass, the time when we sampled leaf traits. The decline in LeafG as a measure of relative chlorophyll concentrations probably goes along with changes in N_Leaf_ as both were usually highly correlated and functionally coupled in leaves (Evans 1989). Increasing SLA at higher species richness has been reported from various biodiversity experiments (Gubsch et al. 2011; Siebenkäs et al. 2017; Bachmann et al. 2018; Pichon et al. 2022) and was explained by the taller canopy height and lower light availability in the canopy of more diverse plant communities (Bachmann et al. 2018). In the Trait‐Based Biodiversity Experiment, it has been shown that plant diversity effects on canopy height were not consistent between seasons and species pools (Guimarães‐Steinicke et al. 2019), which could be a reason that we did not find increased SLA with increasing plant diversity in this experiment. Thus, our results suggest that different leaf traits associated with LES responded to different environmental drivers, which were not consistently coupled with changes in plant diversity.

### 4.2 Leaf Traits in Relation to Species Differences in Spatial and Temporal Resource Acquisition

Leaf traits of the LES were not used to derive trait dimensions among species in the design of the Trait‐Based Biodiversity Experiment because the traits incorporated for designing the experiment were deliberately chosen to represent differences in size‐related spatial and phenology‐related temporal resource acquisition (Ebeling et al. 2014). Our analyses showed that species with different size‐related spatial resource acquisition traits also differed in leaf traits: “large” species had leaf traits associated with a “fast” strategy, that is, low values of LDMC and high values of N_Leaf_ and LeafG, while “small” species showed the opposite characteristics. The only exception was SLA, which did not show significant relationships with the spatial resource acquisition gradient. In contrast to our results obtained with the local species pool, analyses at a global scale showed that size‐related traits (plant height, seed mass) and traits of the LES form two independent major axes of trait variation in a worldwide plant trait space (Price et al. 2014; Díaz et al. 2016), representing a globally larger spectrum of plant size and leaf structure combinations. One possible explanation for the relationships between the gradient in spatial resource acquisition traits (i.e., the scores of PC1 in the PCA used for the design of the Trait‐Based Biodiversity Experiment) and traits of the LES in our experiment is that this axis mainly spanned along a gradient from grasses (low scores on PC1) to forbs (high scores on PC1) in our pool of 48 plant species (Ebeling et al. 2014). Grasses and forbs have been shown to differ in traits related to LES, such as LDMC and N_Leaf_ (Reich et al. 2003; Al Haj Khaled et al. 2005; Siebenkäs et al. 2015; Bachmann et al. 2018) as it is also reflected in the close relationship between the spatial resource acquisition gradient (scores of PC1) and leaf traits in our experiment.

### 4.3 Main Axes of Leaf Trait Variation

Several recent studies from different ecosystem types have indicated that relationships between leaf traits are not “universal” and may vary with the considered scale (Funk and Cornwell 2013; Messier et al. 2017; Anderegg et al. 2018; Buchmann et al. 2018), suggesting a weak coordination of leaf traits in their responses to environmental variation. According to the LES, we expected that leaves possess trait combinations along a gradient from “fast” (low LDMC, high SLA, high N_Leaf_) to “slow” (high LDMC, low SLA, low N_Leaf_) and that this gradient would be reflected along the first axis of principal components analyses based on the measured leaf traits. Our between‐species analysis produced similar leading axes of trait variation for the three species pools and both seasons; however, they only partly fulfilled expectations on trait–trait relationships under the LES: in all species pools, LDMC was opposed to N_Leaf_ as expected according to LES, while SLA showed more variable positions in the trait space.

In contrast, at the within‐species level, that is, analyses focusing on deviations of trait values from species means dependent on plant diversity, we found mostly negative LDMC‐SLA relationships. However, relationships between leaf structure (SLA, LDMC) and N_Leaf_ were more variable. Often, N_Leaf_ was positioned orthogonal to SLA and LDMC, but some species also showed the expected positive relationships between SLA and N_Leaf_ (5 out of 24 cases in each season). These results are in line with an overview of studies summarized in Messier et al. (2017) showing that LDMC‐SLA correlations are common at a local scale, while variation in leaf structure and N_Leaf_ are decoupled from each other. Apart from the “classical” traits included in the LES, we studied leaf greenness (LeafG) as it had been shown to correlate well with chlorophyll concentrations in grassland species (Bachmann et al. 2018). As expected, LeafG was often (but not always) closely related to N_Leaf_ in multi‐trait analyses between species as well as within species.

Overall, our results clearly showed that relationships between leaf traits of the LES are not universal, but are modulated by trait trade‐offs and varying responses of leaf traits to plant diversity at the local scale. Because of the strong context‐dependency of trait–trait relationships within the LES, they cannot be easily used to describe growth and resource allocation strategies of plant species in local plant communities, limiting the general application of the LES.

### 4.4 Extent of Intraspecific Variation in Leaf Traits

Our analyses about the extent of intraspecific trait variation clearly showed that differences in CWM were mainly attributable to between‐species trait differences; that is, they depended on species composition and relative abundances in a community. Nevertheless, ITV contributed 8 to 43% of variation in CWM, which was in the range observed in a global meta‐analysis (e.g., Siefert et al. 2015). The global analysis as well as previous analysis in experimental grasslands (Pichon et al. 2022) reported similar levels of ITV for different LES traits. However, in our study, the contribution of ITV in explaining variation in CWM was by far the lowest for LDMC, although ITV quantified as CV was not much lower than for SLA or LeafG. This indicated that differences between species were more pronounced in LDMC than in other traits. On the contrary, N_Leaf_ had the highest CV and the largest proportion of variation in CWM explained by ITV, showing that this trait responded most sensitively to varying plant communities. Greater ITV of SLA in the pool combining species with different traits in spatial resource acquisition and in “small” than in “large” species (for SLA and LeafG) could be attributable to their plastic responses to varying light conditions, dependent on if they were growing together with other small species or were shaded by taller species (Roscher et al. 2011; Bachmann et al. 2018).

### 4.5 Leaf Traits in Relation to Species Biomass Proportions

According to the leaf‐level trade‐offs of the LES, we expected that species with leaf traits associated with a “fast” strategy were more likely to reach higher biomass proportions in the mixtures than species with a “slow” strategy. Indeed, our hypothesis was fully confirmed by pool SpatRes, for which species with higher biomass proportions in mixtures had lower values of LDMC and higher values of SLA, N_Leaf_ and LeafG than species with lower biomass proportions. Given the correlation of these traits with the spatial‐resource acquisition gradient (STG), these results imply that “large” species with “fast”‐strategy leaves reached higher biomass proportions than “small” species with “slow”‐strategy leaves in mixtures of the pool SpatRes. In the pools TempRes and MixRes, our hypothesis was only partly confirmed: species with higher biomass proportions in mixtures had consistently higher SLA than subordinates. Dominants in plant mixtures of the TempRes pool also had larger values than subordinates in leaf traits associated with the nutritional status (N_Leaf_, LeafG), while this was not the case in the MixRes pool. In a similar analysis, Siebenkäs et al. (2017) also found that traits among dominant and subordinate species did not always differ as expected based on the assumptions of the LES. In their study, dominants also had greater LeafG, while N_Leaf_ did not differ and SLA was even smaller in dominant than in subordinate species. These differences among species pools in our experiment and compared to other experimental studies suggested that the association between species biomass proportions and leaf traits was controlled by the local species pool and the low coordination among leaf traits of the LES.

## 5 Conclusions

In summary, we found that the relationships expected according to the globally described LES did not consistently occur among and within species when growing in local communities of varying plant diversity. Traits associated with the LES retained their ability to show a different extent of ITV and to respond differently to drivers of ITV at the local scale. Therefore, their interpretation as indicators of resource acquisition–conservation trade‐off needs careful consideration at the local scale, limiting the general applicability of the LES.

## Author Contributions

**Dörte Bachmann:** conceptualization (equal), data curation (lead), formal analysis (equal), investigation (equal), writing – original draft (equal), writing – review and editing (supporting). **Nina Buchmann:** conceptualization (equal), funding acquisition (lead), project administration (lead), writing – review and editing (equal). **Anne Ebeling:** investigation (equal), writing – review and editing (equal). **Christiane Roscher:** conceptualization (equal), formal analysis (equal), investigation (equal), writing – original draft (equal), writing – review and editing (equal).

## Conflicts of Interest

The authors declare no conflicts of interest.

## Supporting information


**Data S1:** ece372013‐sup‐0001‐supinfo.docx.

## Data Availability

All data, detailed information about the design of the experiment and the R code are publicly available in the Jena Experiment database (https://jexis.idiv.de/): doi.org/10.25829/6F3Y‐3F71; doi.org/10.25829/swtt‐wc32; doi.org/10.25829/GZY3‐NH61; doi.org/10.25829/4B7H‐FW98; doi.org/10.25829/dj3z‐2e93.
