# Supplementary material for: Traits of the Leaf Economics Spectrum Do Not Always Relate to Species Biomass Proportions in Grassland Communities of Varying Diversity
Source: Ecol Evol. 2025 Sep 11;15(9):e72013. doi: 10.1002/ece3.72013 (PMC12423633; doi:10.1002/ece3.72013)

**Supplementary Material**

**Table S1** Study species including their assignment to experimental species pools spanning gradients in spatial resource acquisition (SpatRes), in temporal resource acquisition (TempRes), or both spatial and temporal resource acquisition (MixRes), their number of occurrences in communities of these pools, and the species positioning along the axes of spatial and temporal resource acquisition (Ebeling et al. 2014)

| Species | Family | SpatRes | TempRes | MixRes | PC1 | PC2 |
| --- | --- | --- | --- | --- | --- | --- |
| *Anthoxanthum odoratum* L. | Poaceae |  | 14 | 14 | -0.743 | -0.966 |
| *Anthriscus sylvestris* (L.) Hoffm. | Apiaceae |  |  | 16 | 1.461 | -0.716 |
| *Centaurea jacea* L. | Asteraceae | 14 |  |  | 1.080 | -0.104 |
| *Cirsium oleraceum* (L.) Scop. | Asteraceae |  |  | 16 | 1.688 | 1.927 |
| *Dactylis glomerata* L. | Poaceae |  | 16 |  | -0.147 | 0.163 |
| *Festuca rubra* L. | Poaceae | 14 |  |  | -1.497 | 0.813 |
| *Geranium pratense* L. | Geraniaceae |  | 14 |  | -0.122 | 1.311 |
| *Glechoma hederacea* L. | Lamiaceae |  |  | 15 | -0.704 | -1.052 |
| *Helictotrichon pubescens* (Huds.) Pilg. | Poaceae | 15 |  |  | -0.904 | -0.026 |
| *Holcus lanatus* L. | Poaceae |  | 14 |  | -0.801 | 0.880 |
| *Knautia arvensis* (L.) J.M. Coult. | Caprifoliaceae | 15 |  |  | 0.972 | 0.381 |
| *Leucanthemum vulgare* (Vaill.) Lam. | Asteraceae | 16 | 16 |  | -0.059 | -0.642 |
| *Phleum pratense* L. | Poaceae | 16 | 15 |  | -0.993 | 0.632 |
| *Plantago lanceolata* L. | Plantaginaceae | 16 | 16 |  | 0.158 | -0.429 |
| *Poa pratensis* L. | Poaceae | 14 |  |  | -1.930 | 0.564 |
| *Prunella vulgaris* L. | Lamiaceae |  |  | 14 | -0.825 | 1.158 |
| *Ranunculus acris* L. | Ranunculaceae |  | 15 |  | 0.055 | -1.022 |
| *Rumex acetosa* L. | Polygonaceae |  |  | 16 | 1.260 | -0.569 |
| *Sanguisorba officinalis* L. | Rosaceae |  |  | 15 | 2.080 | 1.146 |
| *Veronica chamaedry*s L. | Plantaginaceae |  |  | 14 | -1.995 | 1.074 |

**Figure S1** Leaf dry matter content (LDMC) (a), specific leaf area (SLA) (b), leaf nitrogen concentration (N_Leaf_) (c), and leaf greenness (LeafG) (d) related to the temporal trait resource acquisition gradient. Different symbols indicate species assignment to the experimental species pools (see Table S1) with darker colors for measurements in spring and lighter colors for measurements in summer. Relationships were analyzed with linear mixed-effects models (Table 1).


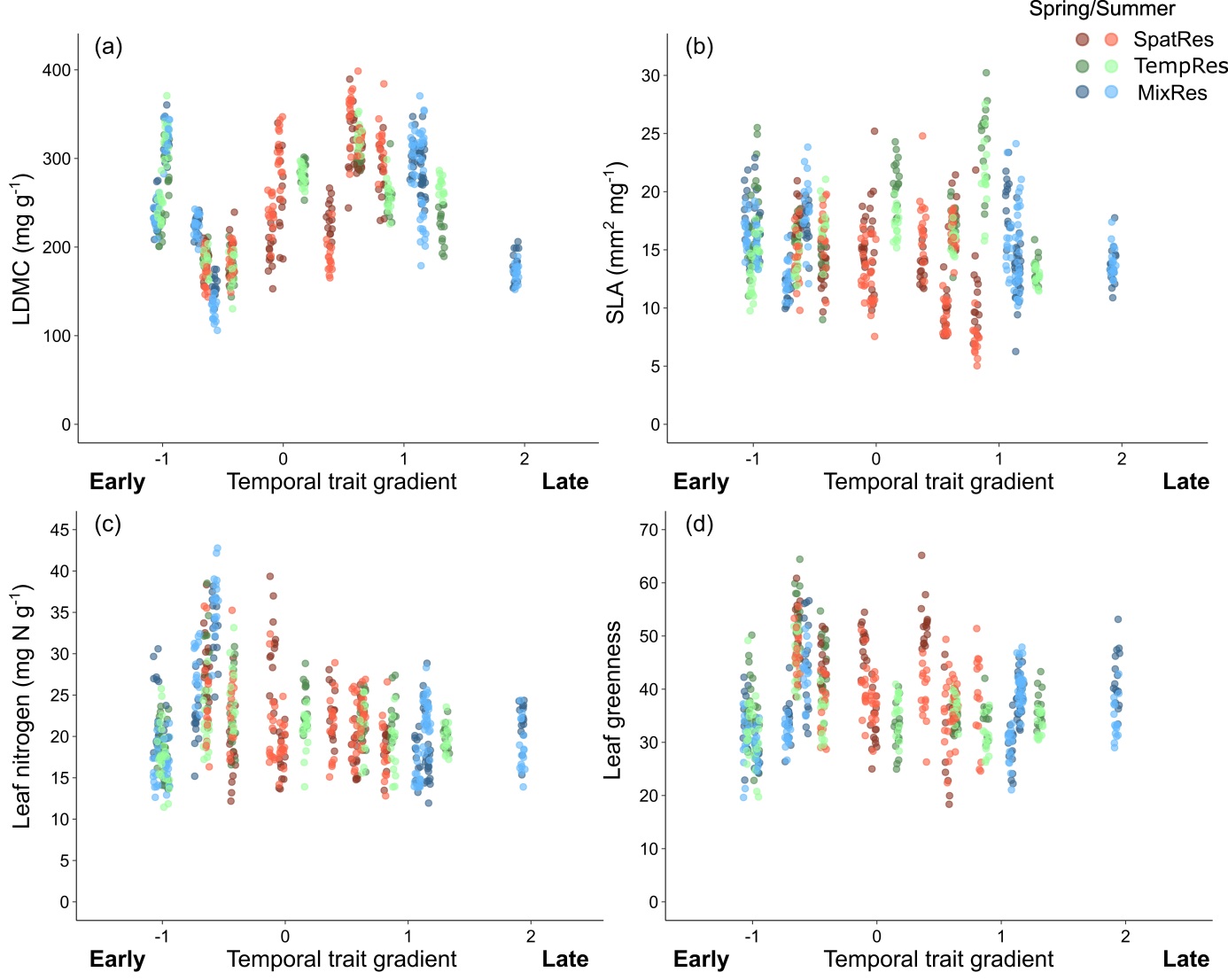


**Figure S2** Standardized principal component analyses (PCA) of leaf traits measured in summer based on species mean traits across all communities (a-c), based on deviations of species trait means per diversity level from species trait means across all communities (d-f), and based on deviations of plot-level species trait values from species trait means per diversity level (g-i). PCA were performed separately for each pool SpatRes (a, d, g), TempRes (b, e, h) and MixRes (c, f, i). Correlation circles based on the first two principal components and proportions of explained variation are given.


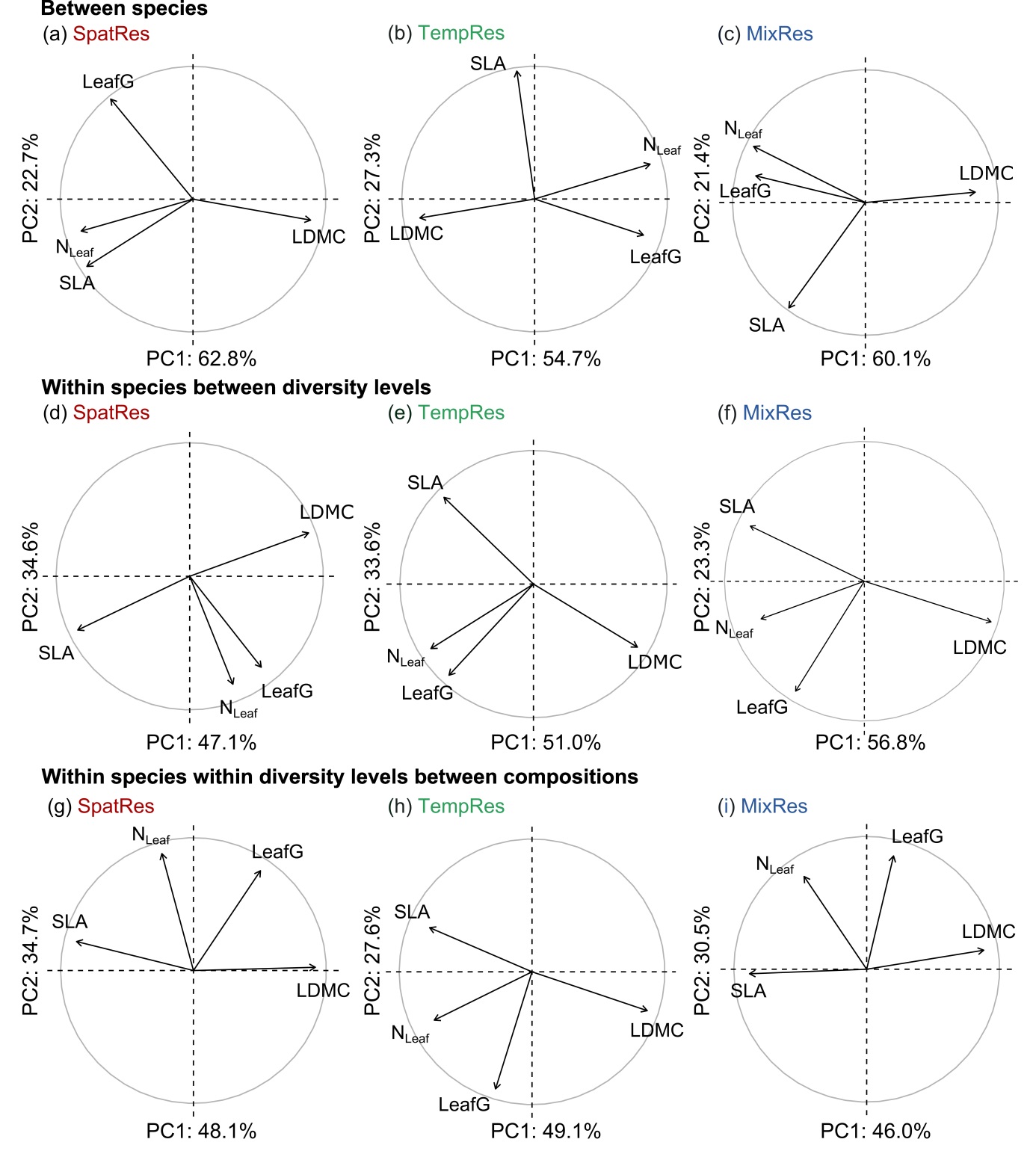


**Figure S3** Standardized principal component analyses (PCA) of leaf traits measured in spring for each species across all communities. Correlation circles based on the first two principal components and proportions of explained variation are given. Species in (a-h) represent the pool SpatRes, species in (i-p) represent the pool TempRes, and species in (q-x) represent the pool MixRes (see Table S1). Note that species which belong to different pools were analyzed separately for their occurrences in each pool. Leaf greenness was excluded in analysis of *F. rubra* because it could not be measured in spring at the narrow leaves of this species.


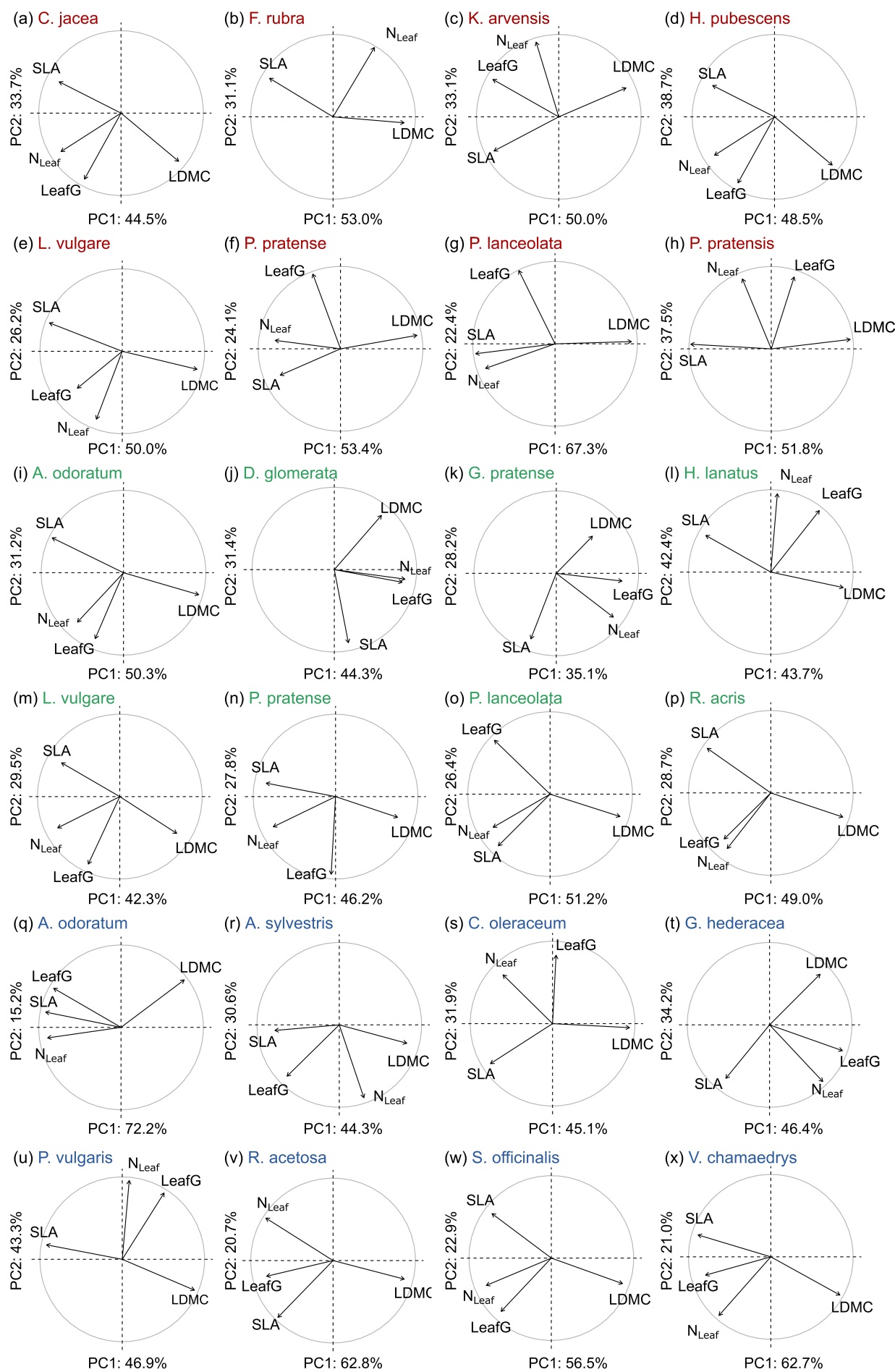


**Figure S4** Standardized principal component analyses (PCA) of leaf traits measured in summer for each species across all communities. Correlation circles based on the first two principal components and proportions of explained variation are given. Species in (a-h) represent the pool SpatRes, species in (i-p) represent the pool TempRes, and species in (q-x) represent the pool MixRes (see Table S1). Note that species which belong to different pools were analyzed separately for their occurrences in each pool.


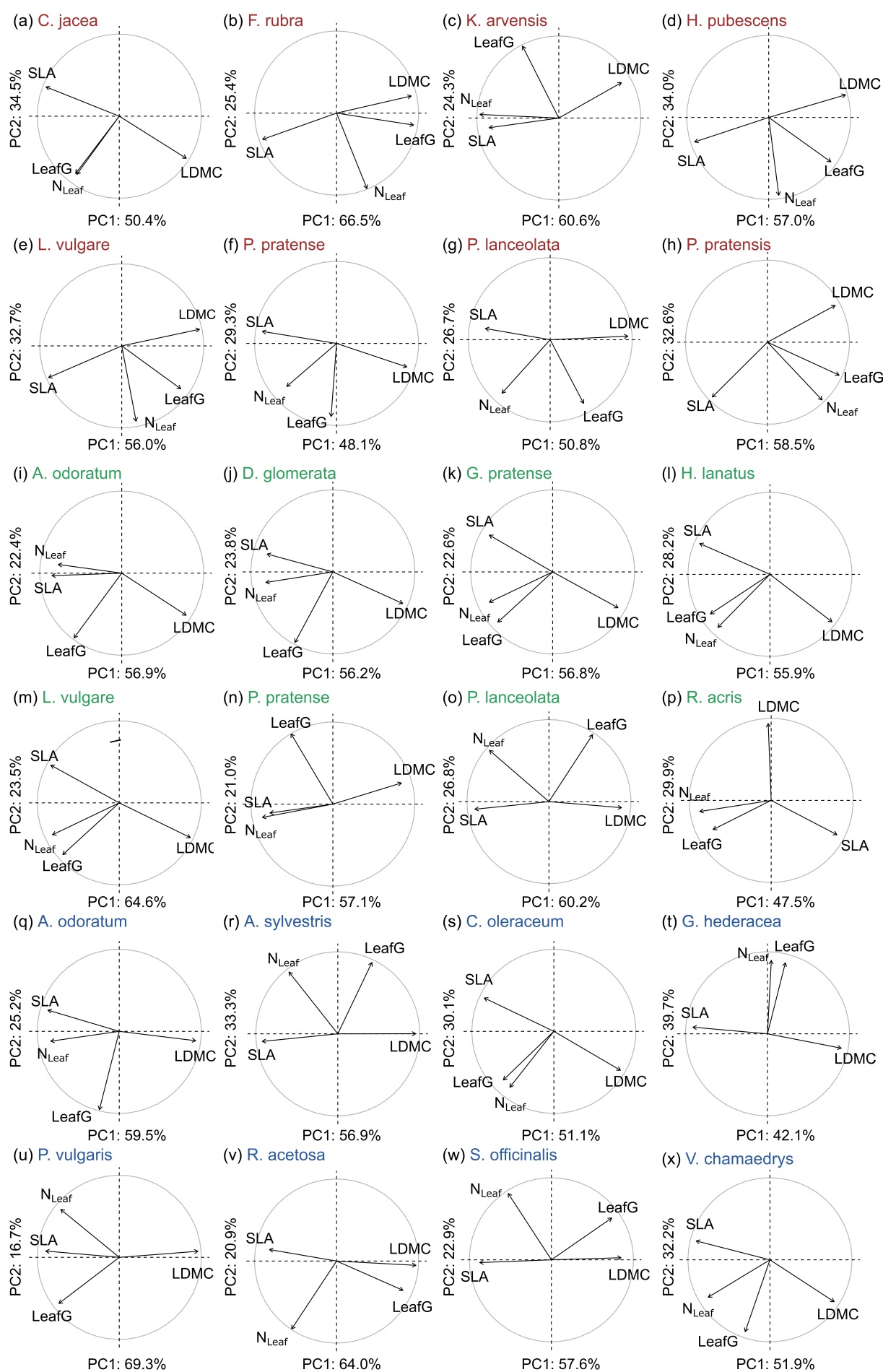


**Figure S5** Intraspecific trait variation (ITV) quantified as coefficient of variation (CV) for each species based on all samples per season. Shown are boxplots for each species pool (SpatRes, TempRes, MixRes) for leaf dry matter content (LDMC) (a), specific leaf area (SLA) (b), leaf nitrogen concentration (N_Leaf_) (c), and leaf greenness (LeafG) (d). Boxes show the median, the interquartile range, maximum and minimum value and outliers. Black dots indicate mean values per species pool.


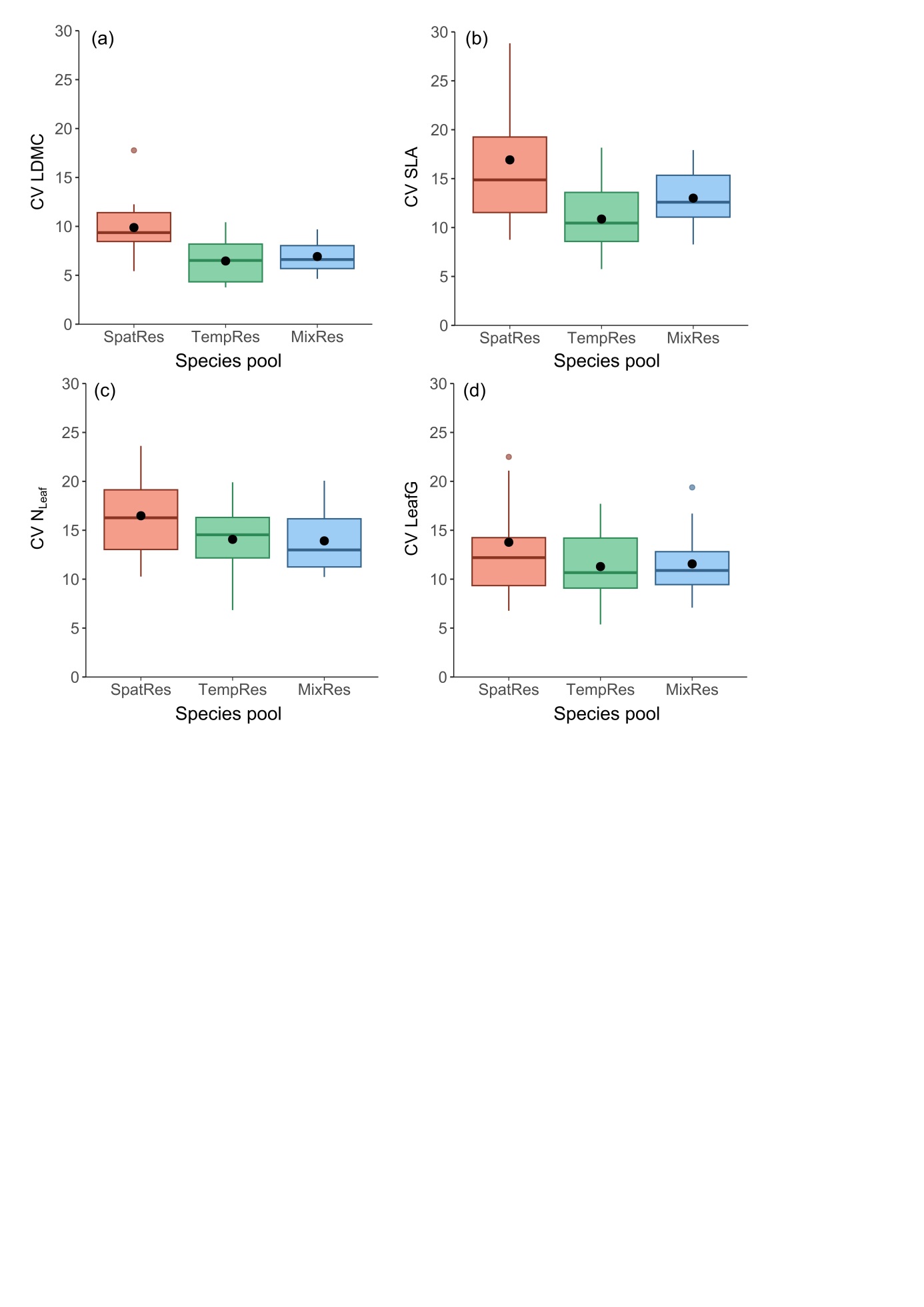

Supplement: Supplementary file 1 — Data S1: ece372013‐sup‐0001‐supinfo.docx. [file ECE3-15-e72013-s001.docx]
